# TALEN-mediated homologous recombination in *Daphnia magna*

**DOI:** 10.1038/srep18312

**Published:** 2015-12-17

**Authors:** Takashi Nakanishi, Yasuhiko Kato, Tomoaki Matsuura, Hajime Watanabe

**Affiliations:** 1 Department of Biotechnology, Graduate School of Engineering, Osaka University, 2-1 Yamadaoka, Suita, Osaka, Japan; 2 Frontier Research Base for Global Young Researchers, Graduate School of Engineering, Osaka University, 2-1 Yamadaoka, Suita, Osaka, Japan

## Abstract

Transcription Activator-Like Effector Nucleases (TALENs) offer versatile tools to engineer endogenous genomic loci in various organisms. We established a homologous recombination (HR)-based knock-in using TALEN in the crustacean *Daphnia magna*, a model for ecological and toxicological genomics. We constructed TALENs and designed the 67 bp donor insert targeting a point deletion in the *eyeless* mutant that shows eye deformities. Co-injection of the TALEN mRNA with donor DNA into eggs led to the precise integration of the donor insert in the germ line, which recovered eye deformities in offspring. The frequency of HR events in the germ line was 2% by using both plasmid and single strand oligo DNA with 1.5 kb and 80 nt homology to the target. Deficiency of *ligase 4* involved in non-homologous end joining repair did not increase the HR efficiency. Our data represent efficient HR-based knock-in by TALENs in *D. magna*, which is a promising tool to understand *Daphnia* gene functions.

Water fleas, *Daphnia* spp., are small planktonic crustaceans ubiquitously found in fresh water environments. Because of their important position in the aquatic food chain and highly plastic phenotypes in response to environmental changes, they have been used as model organisms in ecology and toxicology for decades^1^,^2^. For instance, *Daphnia* propagates quickly by parthenogenesis in an environmentally rich condition, whereas it switches reproductive mode from asexual to sexual in response to environmental degradation such as food limitation and/or short photoperiods. To reveal the molecular basis of these unique phenotypes, the ESTs and whole genome have been sequenced (*D. magna*^3^,^4^ and *D. pulex*^5^) and transcriptome analyses have been conducted^6^,^7^,^8^. Hence, to clarify the link between phenotypes and accumulated genetic information, efficient genetic engineering techniques are necessary.

Recently, we succeeded in the transgenesis of *D. magna* via random DNA integration, which allowed us to observe *Daphnia* embryogenesis by using fluorescent proteins^9^. However, the random DNA integration method has three major problems: low efficiency (less than 1% in total fertile adults), probable partial deletion of genes of interest, and an untargetable nature. To address these problems, we focused on the homologous recombination method, which allowed researchers to precisely integrate genes of interest into a targeted genomic site^10^,^11^,^12^. In the present study, we applied this method to *D. magna* by using programmable nucleases, as the homologous recombination with such nucleases was reported to be efficient in various organisms^13^,^14^,^15^,^16^,^17^,^18^,^19^,^20^,^21^.

Programmable nucleases are promising tools to induce targeted gene knock-out and knock-in^22^,^23^. They can be divided into two categories: custom-designed artificial nucleases such as Zinc Finger Nucleases (ZFNs) or Transcription Activator-Like Effector Nucleases (TALENs), and RNA-guided nucleases based on Clustered Regularly Interspaced Short Palindromic Repeats (CRISPR)/CRISPR-associated (Cas) system. Both ZFN and TALEN are fusion proteins, which consist of a programmable DNA binding domain and a *Fok*I nuclease domain and act as a dimer to induce double strand breaks (DSBs) into a target genomic site. However, the CRISPR/Cas system requires two components: programmable single guide RNAs (sgRNAs) and Cas9 nucleases. sgRNAs are designed to be complementary to target genome sequences. Cas9 nucleases induce DSBs at a target site under the guidance of sgRNAs. The broken site is mainly repaired through either Non-Homologous End Joining (NHEJ) or Homologous Recombination (HR) pathways. The NHEJ repair often leads to incorporation of random insertions or deletions (in-dels), which results in knocking out of a targeted gene by frame shift mutations. However, the HR repair leads to precise integration of foreign genes via HR between genome and donor DNA including genes of interest. Depletion of NHEJ components such as Ligase4 or Ku70 leads to a significant increase of HR efficiency in various organisms^13^,^24^,^25^,^26^,^27^. Recently, we proved that both TALEN and CRISPR/Cas systems could be applied to *D. magna* to induce the targeted gene knock-out via NHEJ^28^,^29^.

In the current study, HR-mediated integration of exogenous DNA using TALENs was tested. We succeeded in the precise integration of 67 bp of exogenous DNA into the targeted genomic locus at around a 2% ratio by co-injection of TALEN mRNAs and donor DNAs. Moreover, we confirmed the availability of two types of donor DNAs—plasmid DNA and single stranded oligo DNA (ssODN). Compared to the random DNA integration method, the TALEN-mediated HR method showed high efficiency with a precise and targetable nature. To achieve further efficient HR knock-in via TALEN, we disrupted the *Daphnia magna-ligase 4* (*Dma-lig4*) gene. However, enhancement of HR knock-in efficiency was not observed in this *Dma-lig4* deficient strain. Overall, our data showed that the TALEN-mediated HR method can be applied to *D. magna*, and this method will be beneficial for future analyses of *Daphnia* gene functions.

## Results

### Strategy to test the activity of TALEN-mediated HR

**Figure 1:**
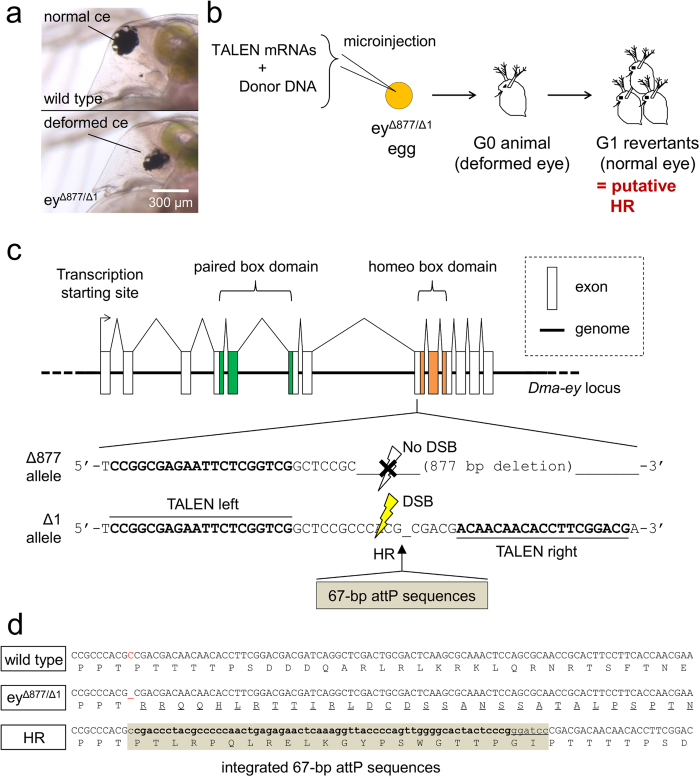
Strategy to induce TALEN-mediated HR in *D. magna.* (**a**) Typical deformed eye phenotype of the ey^Δ877/Δ1^ strain. The top picture is derived from the wild type with a normal spherical compound eye (ce). The bottom picture is derived from the ey^Δ877/Δ1^ strain with deformed ce. Scale bar indicates 300 μm. (**b**) Overview of screening HR daphniids by phenotypic rescue. TALEN-mediated HR was induced by co-injection of TALEN mRNAs and donor DNA carrying exogenous DNA fragment into eggs from the ey^Δ877/Δ1^ strain. Because the integration of exogenous DNAs via HR was designed to result in recovering the frameshift mutation on the Δ1 allele in *Dma-ey* locus (see Fig. 1c,d), it was expected that the deformed eye phenotype would be rescued by germ line transmission of HR events. (**c**) Schematic gene structure of *Dma-ey* and partial sequences on the Δ877 allele and Δ1 allele. The *Dma-ey* gene putatively consists of 13 exons (shown by open boxes). DNA-binding domain-encoding regions are coloured in green (paired box) and orange (homeo box). The ey^Δ877/Δ1^ strain has 877 bp or 1 bp deletion mutations on respective alleles of the *Dma-ey* locus. TALEN-binding DNA sequences are highlighted in bold. TALEN proteins can induce double strand breaks (DSBs) only into the Δ1 allele because of the long deletion on the Δ877 allele, so that 67-bp attP would be integrated into the Δ1 allele via HR. (**d**) Comparison of putative genome sequences around the TALEN targeted sites among the wild type, ey^Δ877/Δ1^ strain, and HR revertant daphniids. Deduced amino acid sequences of each strain are described under the genome DNA sequence. The deleted base ‘C’ on the ey^Δ877/Δ1^ genome is highlighted in red. Altered Dma-EY peptide sequences are indicated by underline in ey^Δ877/Δ1^ strain. The entire integrated 67-bp fragment, which consists of 1 base of cytosine, 60 bases of attP (bold), and 6 bases of the *Bam*HI recognition site (underline) is coloured beige.

To easily test if the TALEN-mediated HR is successful or not in *D. magna*, we needed to target a gene that shows a screenable phenotype. In our previous study, we found that biallelic mutants of *Daphnia magna eyeless* (*Dma-ey*, ortholog of mammalian *pax6*) gene showed compound eye deformity which is clear in appearance compared with the wild type^28^,^29^ (Fig. 1a). Thus, we designed phenotypic rescue experiments via TALEN-mediated HR as shown in Fig. 1. In this strategy, TALEN mRNAs are co-injected into the biallelic *Dma-ey* mutant eggs with donor DNA carrying exogenous DNA fragments to repair the mutation. If TALEN-mediated HR is successfully induced, the eye phenotype of the biallelic mutant is recovered, which will allow us to test the activity of TALEN-mediated HR (Fig. 1b).

To realise the above strategy, we designed an exogenous DNA fragment to rescue the deformed eye phenotype of the viable biallelic *Dma-ey* mutant^28^, named as ey^Δ877/Δ1^ in the present study, which has the 877 bp deletion on one of the *Dma-ey* alleles (=Δ877 allele) and the 1 bp deletion on another allele (=Δ1 allele) (Fig. 1c). The Δ1 allele was chosen as a target site of HR. To correct the *Dma-ey* reading frame, we designed the 67 bp DNA fragment containing attP that is the target DNA sequence of phiC31 integrase^30^ (Fig. 1c,d). This DNA fragment consisted of 1 base of cytosine, 60 bases of the core attP, and 6 bases of *Bam*HI recognition site in the 5′ to 3′ direction on the sense strand (Fig. 1d). The 67 bp in-frame insertion would result in adding 22 peptides into Dma-EY proteins in the linker region between the paired box and homeobox domains (Fig. 1d). Therefore, we hypothesised that the 67 bp insertion would have no adverse effect on Dma-EY functions and the deformed eye phenotype would be rescued.

### Design of TALEN proteins

First, a heterodimeric TALEN pair to induce double strand breaks (DSBs) into the Δ1 target site was constructed (Fig. 1c). TALEN assemblies of the RVD-containing repeats were performed using the Golden Gate approach^31^. As the TALEN-right binding site on the Δ877 allele is destroyed, heterodimeric TALEN proteins can exclusively target the Δ1 allele (Fig. 1c). To evaluate the activity of newly constructed TALENs to induce DSBs in the target site of *Dma-ey*, 125, 250, and 500 ng/μL of each TALEN pair mRNAs were injected into wild type eggs. As a result, the compound eye deformity was observed at 28, 22, and 42% of survived juveniles, respectively (Table 1a). Therefore, we concluded that the new TALEN pair was effective and the 500 ng/μL mRNA concentration was optimal among tested conditions.

### Design of donor DNAs

Then, we designed donor DNAs required for inducing HR. Following recent studies that described that plasmid DNAs and ssODNs could be used as donor DNAs^22^,^23^, we constructed 3 types of donor DNAs: one targeting plasmid DNA with a total of 1.5 kb homology to the target site, and two targeting ssODNs with a total of 40 or 80 nt homology (Supplementary Data S1). All 3 donor DNAs had the common 67 bp of the exogenous attP fragment described above. The existence of the 67 bp insert into the donor DNAs would interrupt TALEN cleavages on donor DNAs, so that TALEN proteins should only cut the target on the chromosome.

In terms of toxicity for injecting DNAs, we previously found that the injection of around 50 ng/μL plasmid DNA does not show severe toxicity to injected embryos^9^. However, the toxicity of ssODN in *D. magna* has not been tested. Therefore, the shorter donor ssODN was injected into wild type eggs to check its toxicity. When we injected 30, 100, and 300 ng/μL ssODNs, the survival rate of injected daphniids were 74, 45 and 0%, respectively (Table 1B). Taken together, we chose 50 ng/μL plasmid DNAs and 100 ng/μL ssODNs for the TALEN-mediated HR experiments.

### Co-injection of TALEN mRNAs and targeting plasmid DNA

**Figure 2:**
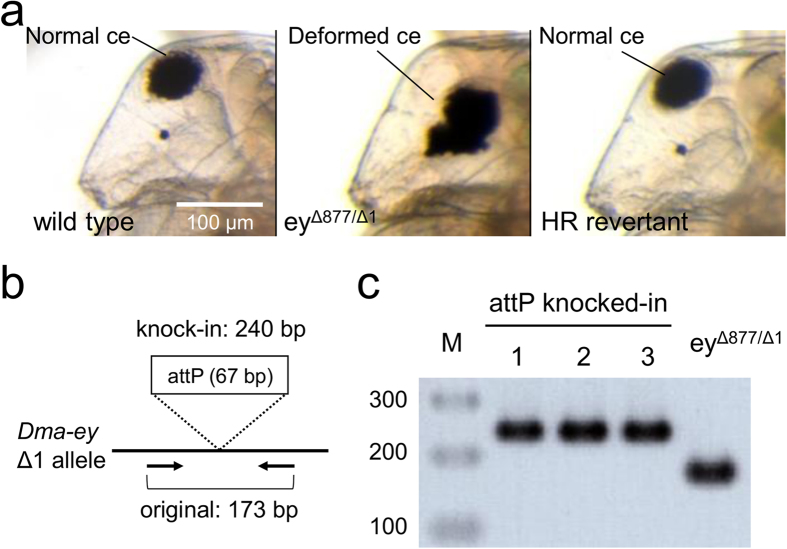
Comparison of phenotypes and genotypes between parental ey^Δ877/Δ1^ strain and HR revertants. (**a**) Lateral pictures of wild type (left), parental ey^Δ877/Δ1^ strain (centre), and HR revertant (right). The ey^Δ877/Δ1^ strain shows the deformed, non-spherical compound eye (ce) phenotype. The HR revertant shows the normal eye phenotype and wild type strain. Scale bar indicates 100 μm. (**b**) Schematic illustration of genomic PCR to check the integration of attP into the *Dma-ey* Δ1 allele. Black thick bar indicates the *Dma-ey* Δ1 allele. Primers are shown by arrows. The attP knock-in results in 240 bp long PCR products, whereas no modification gives 173 bp long PCR products. (**c**) Agarose gel electrophoresis of genomic PCR products using HR revertant strains. 1 and 2 are derived from co-injection of TALEN mRNAs and targeting plasmid, whereas 3 is derived from co-injection of TALEN mRNAs and ssODN with 80 nt homology. attP knocked-in HR revertants showed 240 bp PCR products, whereas parental ey^Δ877/Δ1^ strain showed 173 bp PCR products. M indicates the marker DNA.

To test whether HR can be induced by TALEN with circular donor DNA in *D. magna*, TALEN mRNA and targeting plasmid DNAs were co-injected into eggs from the ey^Δ877/Δ1^ strain. 101 adults (called Generation 0: G0) were obtained from 135 injected eggs (75% survival, Table 2) in three times replicated experiments (details in Supplementary Table S1). Since daphniids propagate by parthenogenesis in favourable conditions, each of the injected G0 adults produced G1 offspring without mating. The eye phenotypes of G1 offspring from each G0 mother were observed microscopically. It was found that 8 out of 101 G0 mothers produced G1 revertant daphniids showing the normal eye phenotype (Table 2, Fig. 2a).

To check if normal eye G1 offspring had attP insertions into the targeted Δ1 allele, genomic PCR was performed using primers to amplify the integration site. Based on our experimental design, attP integration should give 240 bp-long PCR products, whereas no modification gives 173 bp-long PCR products (Fig. 2b). As a result, 2 out of 8 revertant G1 lines showed the 240 bp PCR products. In addition, DNA sequencing analysis proved that a single copy of attP was precisely integrated into their genomes. (Figs 1d and 2c: attP knocked-in 1 and 2, Table 2). However, the other 6 lines had additional in-del mutations, which corrected the reading frame of *Dma-ey* gene (Supplementary Data S2). In summary, the TALEN-mediated HR in germ line genomes occurred in 2 out of 101 fertile G0 adults (about 2% efficiency, Table 2). The integrated attP fragment on genomes was stably inherited by more than 10 generations.

### Co-injection of TALEN mRNAs and ssODNs

Next, to test the availability of ssODN as the donor DNA to induce TALEN-mediated HR in *D. magna*, TALEN mRNAs and either ‘short’ ssODNs with 40 nt homology or ‘long’ ssODNs with 80 nt homology were co-injected into eggs from the ey^Δ877/Δ1^ strain. For short ssODNs, 88 G0 adults were obtained from 147 injected eggs (60% survival, Table 2) in four times replicated experiments (details in Supplementary Table S1). 7 out of 88 G0 mothers produced G1 revertant daphniids. However, none of the 7 G1 lines had attP sequences at the targeted Δ1 allele but had various additional in-dels there instead (Supplementary Data S2). In the case with long ssODNs, 52 G0 adults were survived from 98 injected eggs (53% survival, Table 2) in one experiment (details in Supplementary Table S1). 8 out of 52 G0 mothers produced normal eye G1 offspring, and 1 out of the 8 G1 lines had the precise insertion of attP (Figs 1d and 2c: attP knocked-in 3, Table 2). Taken together, TALEN-mediated HR occurred in 1 out of 52 fertile G0 adults (about 2% efficiency, Table 2) using ssODNs with longer homology arms. Thus, we proved the availability of ssODN for TALEN-mediated HR knock-in in *D. magna*.

### Establishment of the *lig4* deficient strain by CRISPR/Cas

**Figure 3:**
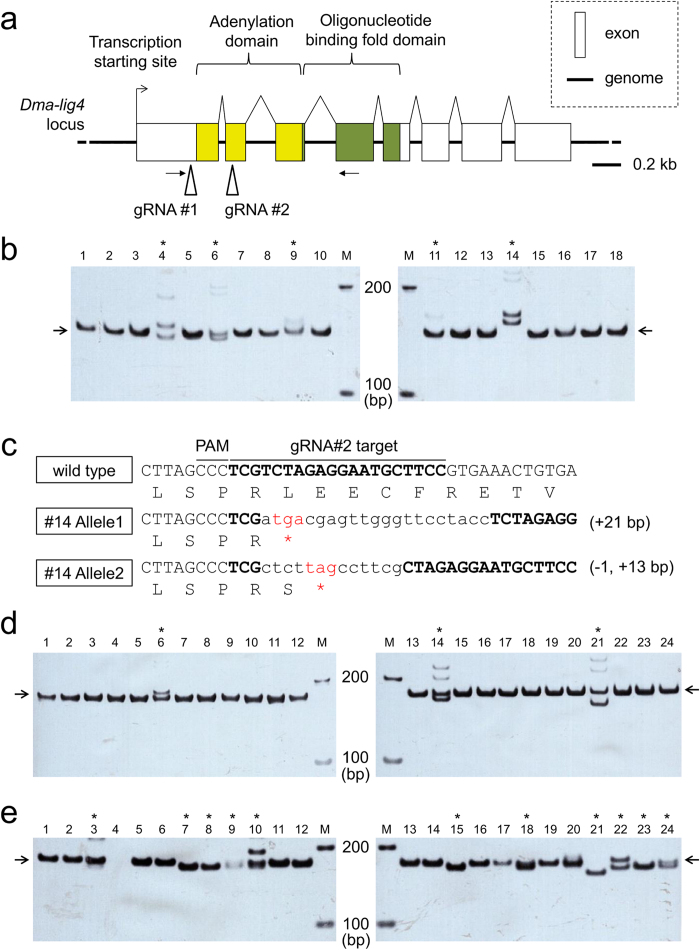
Establishment and use of *Dma-lig4* deficient mutant by CRISPR/Cas. (**a**) Schematic gene structure of *Dma-lig4*. *Dma-lig4* gene is annotated to consist of 8 exons (open boxes). Putative catalytic domain-encoding regions are highlighted in yellow (adenylation domain) and light green (oligonucleotide binding fold domain). gRNA-targeted sites are shown by triangles. Primers used for RT-PCR are shown by black arrows. Scale bar indicates 0.2 kb. (**b**) PAGE analyses around gRNA #2 target site of putative *Dma-lig4* mutants. The genomic region surrounding gRNA #2 target site was amplified from genome DNAs of randomly selected 18 G1 lines. The original size of the PCR products should be 145 bp, indicated by black arrows. Thus, different patterns of band migration indicate the various in-del formation on the gRNA #2 targeted site. Asterisks (*) show the putative monoallelic and/or biallelic mutants. M indicates the marker DNA. (**c**) Genotype of *Dma-lig4* biallelic mutant strain. Deduced amino acid sequences of wild type or *Dma-lig4* mutant #14 are shown under the genome DNA sequences. gRNA #2 targeted sequences and Protospacer Adjacent Motif (PAM) are lined. Especially, gRNA #2 targeted sequences are highlighted in bold. Lower case indicates the introduced in-del mutations into mutant #14 genome. As shown in red, a premature termination codon was introduced into both *Dma-lig4* alleles in the mutant #14 genome. (**d**,**e**) PAGE analyses of G1 offspring from TALEN and targeting plasmid injection using ey^Δ877/Δ1^ strain with or without *Dma-ey* deficiency (d, e, respectively). To estimate the germ line in-del mutation rate, the genomic region surrounding the TALEN targeted site was amplified from genome DNAs of randomly selected 24 G1 lines. Original size of the PCR products should be 173 bp, indicated by black arrows. Thus, different patterns of band migration indicate the various in-del formations on TALEN targeted site. Asterisks (*) show the putative in-del introduced lines. M indicates the marker DNA.

To increase the efficiency of TALEN-mediated HR, we tried to disrupt the *ligase 4* (*lig4*) gene of the ey^Δ877/Δ1^ strain. We searched the *D. magna* genome database and found one ortholog of the *lig4* gene (*Dma-lig4*), which was annotated to consist of 8 exons (Fig. 3a). The predicted 727 amino acids were aligned with human, *Drosophila melanogaster*, and yeast (*Saccharomyces cerevisiae*) DNL4 proteins (Supplementary Data S3). Importantly, all of the 4 domains conserved among the species were found in addition to the active residue lysine at position 175 in the adenylation domain^32^,^33^,^34^ (Supplementary Data S3). The expression of this gene was confirmed by reverse transcribed (RT)-PCR with primers encompassing the adenylation domain (Fig. 3a, Supplementary Data S4) and sequencing of the PCR products, suggesting that the Dma-Lig4 might function in NHEJ pathway (Supplementary Data S1).

Next, to disrupt the *Dma-lig4* gene in the ey^Δ877/Δ1^ strain by CRISPR/Cas system, two guide RNAs (gRNAs) were designed around the adenylation domain (Fig. 3a). *In vitro* synthesised Cas9 mRNAs (500 ng/μL) and two gRNAs (50 ng/μL each) were co-injected into eggs from ey^Δ877/Δ1^ strain. Fifty G0 fertile adults survived from 66 injected eggs. Although *lig4* knock-out leads to lethal phenotypes in mammals, *lig4*-deficient *Drosophila* is viable and showed no sign of abnormal phenotypes^35^,^36^. We expected that the phenotype of *Dma-lig4* deficient daphniids may be similar to that of *Drosophila* and we used G1 daphniids from the survived adults for further analyses.

To obtain homozygous *Dma-lig4* mutants from 50 G1 lines, 18 of them were randomly selected and genotyped at gRNA-targeted *Dma-lig4* loci. Genomic PCR was performed using primers to amplify the gRNA #2 targeted site and polyacrylamide gel electrophoresis (PAGE) indicated that 5 of the 18 G1 lines had various in-del mutations (Fig. 3b). Sequencing of the amplified PCR fragments revealed that the #14 line has premature stop codons on both alleles of *Dma-lig4* (Fig. 3c). This line did not have any mutation at the gRNA #1 targeted site. We used this line as an ey^Δ877/Δ1^ strain with *Dma-lig4* deficient background.

### Application of TALEN-mediated HR to the ey^Δ877/Δ1^ strain with *Dma-lig4* deficiency

First, to check if the NHEJ repair activity is declined in *Dma-lig4* mutants, we compared the germ line in-del mutation rates between the *Dma-lig4* deficient ey^Δ877/Δ1^ strain and the original ey^Δ877/Δ1^ strain. Co-injection of TALEN mRNAs and targeting plasmid DNAs was performed into the *Dma-lig4* deficient ey^Δ877/Δ1^ strain that we established in the above experiments, and 46 G0 adults were obtained from 61 injected eggs (Table 3). Twenty-four of 46 G1 lines were randomly selected and genotyped on the TALEN targeted site. Three of 24 (13%) had in-dels on the targeted genomic site (Fig. 3d). In contrast, co-injection of the same constructs into the original ey^Δ877/Δ1^ strain (see the section ‘Co-injection of TALEN mRNAs and targeting plasmid’) led to in-del formation in 11 of 24 (46%) G1 lines (Fig. 3e), suggesting that the NHEJ repair activity was declined in the *Dma-lig4* deficient ey^Δ877/Δ1^ strain.

Second, to evaluate the efficiency of TALEN-mediated HR in the *Dma-lig4* deficient ey^Δ877/Δ1^ strain, the normal eye revertants obtained from the above injection were genotyped. Of the 46 G1 lines, two normal eye revertant G1 lines were observed (Table 3). However, the genotyping analysis showed that attP was not integrated into their genome DNA, suggesting that the HR efficiency in the *Dma-lig4* deficient strain might be less than 2% (=1/46). Given the 2% HR efficiency in the original ey^Δ877/Δ1^ strain with wild type *Dma-lig4*, depletion of the *Dma-lig4* gene may not have positive effects on TALEN-mediated HR. Although *Dma-lig4* deficiency led to the decline of NHEJ-repair activity, it might not contribute to increased HR efficiency in *D. magna*.

## Discussion

In the present study, we proved that TALEN-mediated precise DNA integration via HR could be achieved in *D. magna*. As in pioneering studies^22^,^23^, both targeting plasmid and ssODN functioned as donor DNA in *D. magna*. The TALEN-mediated HR method showed a high transgenesis efficiency (2% in total fertile adults) and enabled us to precisely integrate a single copy of the gene of interest into a predetermined genomic locus. The germ line transmission efficiency of nuclease-induced HR events in *D. magna* is likely to be similar to those in other animals such as the fly^14^ and zebrafish^20^, or higher than that in mosquitoes^21^,^26^. Thus, this method overcame the major problems of the random DNA integration method as discussed in the introduction section and provides an efficient way to create transgenic daphniids.

The targeted DNA integration method would be useful for further advanced molecular experiments in *D. magna*. In the present study, we adapted the attP, which is the landing site of phiC31 integrase as a model insert DNA, and introduced it into the targeted *Dma-ey* locus. We confirmed that the integrated attP fragment on the *D. magna* genome was stably inherited in more than 10 generations. This attP-carrying strain will be applicable to the phiC31 integrase/attP-attB system in *D. magna* for future research^30^,^37^. Moreover, the TALEN-mediated HR method can be used for the creation of floxed alleles or fusion of genes with epitope tags such as HA or His. This study offers fundamental technology that will enable us to perform further sophisticated molecular experiments on *D. magna* in the future, such as conditional knock-outs via Cre/loxP or chromatin immunoprecipitation assays.

Although we used TALEN as a nuclease in the current experiments, the CRISPR/Cas system would similarly induce HR in *D. magna*. Since the activity of Cas9 nuclease is dependent on targeted sequences of gRNAs^38^,^39^, screening of highly efficient gRNAs should be important to achieve CRISPR/Cas-mediated HR. A recent study showed that high-resolution melt analysis (HRMA) assay-based selection of active gRNAs enabled HR-mediated DNA integration by CRISPR/Cas in mosquitoes^26^. Due to the relative ease and fast construction of CRISPR/Cas-based technology, the CRISPR/Cas-mediated HR method will also provide a simple and efficient way to engineer *Daphnia* genomes.

The TALEN-mediated HR method should be tested for homozygous target alleles. In the present study, we targeted the hemizygous *Dma-ey* Δ1 allele as a recipient genomic locus to integrate the exogenous attP fragment. The transgenesis efficiency in germ cells was around 2%, whereas it is still unclear whether a similar ratio would be observed when targeting homozygous alleles. If the TALEN-mediated HR method also exhibits high efficiency of gene knock-in into a homozygous target gene, we could target a wider range of genes in the *D. magna* genome.

Pioneering works described that the disruption of NHEJ components such as Lig4 or Ku70 led to an increase of HR knock-in efficiency in various animals and plants^13^,^24^,^25^,^26^,^27^. However, our data indicated that *Dma-lig4* deficiency might decrease the NHEJ activity but not improve HR efficiency in *D. magna*. In the case of *Arabidopsis*, a Ku70 knock-out mutant showed higher HR frequency than a Lig4 mutant^25^. Therefore, we may be able to achieve higher HR efficiency by mutating Ku70 genes in *D. magna*.

The results from the present study enable us to analyse the link between genetic information and phenotypes by simple gene disruption and the various technologies discussed above. The TALEN-mediated HR method will contribute to a more comprehensive understanding of the accumulated knowledge of *Daphnia* in ecology and toxicology at the molecular level.

## Methods

### *Daphnia* strains

The wild type *D. magna* strain (NIES clone) was obtained from the National Institute for Environmental Studies (NIES, Tsukuba, Japan) and cultured under laboratory conditions for many generations. The ey^Δ877/Δ1^ mutant strain we previously established^28^ was used in this study. The ey^Δ877/Δ1^ strain with the *Dma-lig4* deficient background was established in this study by CRISPR/Cas-mediated mutagenesis (details in Results section).

### *Daphnia* culture conditions

To minimise variations in maternal effects that may influence microinjection, we maintained the ey^Δ877/Δ1^ strain under the following conditions: 60 neonates (under 24 h) were transferred to 2.5 L of ADaM medium^40^ and cultured at 22–24 °C, under a light/dark photoperiod of 16/8 h. The culture medium was changed once a week. The ey^Δ877/Δ1^ daphniids were fed once a day with 3 × 10^8^ cells of *Chlorella vulgaris* (Nikkai Center, Tokyo, Japan) during the first week; after they matured, their offspring was removed once per day, and they were fed 4 × 10^8^ cells of *Chlorella* daily. The culturing procedure was the same for ey^Δ877/Δ1^ with the *Dma-lig4* deficiency.

### Molecular reagents

#### TALEN expression vectors

The TALEN target sites were selected using TAL Effector Nucleotide Targeter (TALE-NT) 2.0^41^ with the following parameters: (1) spacer length of 15 to 20 nucleotides, (2) repeat array length of 15 to 20 nucleotides, (3) NN for G substitute, (4) T at position 0, and (5) using guidelines proposed by Streubel *et al.*^42^. The TALEN recognition sequences are, left TALEN 5′-CCGGCGAGAATTCTCGGTCG-3′ and right TALEN 5′-CGTCCGAAGGTGTTGTTGT-3′. The spacer between the two binding sites was 18 bp long in the wild type genome, whereas 17 bp long in the ey^Δ877/Δ1^ genome.

To generate TALEN DNA-binding regions, TALEN assemblies of the RVD-containing repeats were conducted using the Golden Gate approach^31^. Once assembled, the RVD-containing repeats were cloned into a pT3TS destination vector with the GoldyTALEN backbone (Addgene plasmid 38142^19^), resulting in construction of pT3TS-ey-KI-TALEN-left and pT3TS-ey-KI-TALEN-right vectors. To construct heterodimeric TALEN expression vectors, pT3TS-ey-KI-TALEN-left and pT3TS-ey-KI-TALEN-right were digested with *Xba*I and *Bsa*BI endonucleases (NEW ENGLAND BioLabs, Connecticut, USA). The digested fragments encoding RVD-containing repeats were cloned into the *Xba*I/*Bsa*BI site of pCS-Dmavas-dsr-TALEN-left-ELD and pCS-Dmavas-dsr-TALEN-right-KKR, both of which were constructed in our previous study^29^, resulting in the construction of pCS-Dmavas-ey-KI-TALEN-left-ELD and -right-KKR, respectively.

#### Cas9 and gRNA expression vectors

The Cas9 expression vector pCS-Dmavas-Cas9 was constructed in our previous study^28^. To generate the gRNA expression vector pDR274-Dma-lig4, the plasmid DR274 (Addgene plasmid 42250^43^) was digested with *Bsa*I (NEW ENGLAND BioLabs, Connecticut, USA), followed by dephosphorylation with Antarctic Phosphatase (NEW ENGLAND BioLabs, Connecticut, USA). A pair of *Dma-lig4* targeting oligonucleotides was annealed and then ligated into the linearized pDR274 vector using a ligation mix (TaKaRa Bio, Shiga, Japan). The genomic target sites and sequences of the oligonucleotides used in this study are listed in Supplementary Table S2.

#### *In vitro* RNA syntheses

For the syntheses of TALEN mRNAs, TALEN expression vectors were linearized with *Acc*65I endonuclease, purified by QIAquick PCR purification kit (QIAGEN GmbH, Hilden, Germany). Linearized DNA fragments were used for *in vitro* transcription with mMessage mMachine kit (Life Technologies, California, USA). Poly (A) tails were attached to TALEN RNAs by using a Poly(A) Tailing Kit (Life Technologies), following the manufacturer’s instructions. The synthesised RNAs were column purified using mini Quick Spin RNA columns (Roche diagnostics GmbH, Mannheim, Germany), followed by phenol/chloroform extraction, ethanol precipitation, and dissolution in DNase/RNase-free water (Life Technologies).

For the syntheses of Cas9 mRNAs, templates with a T7 promoter were amplified by PCR from the pCS-Dmavas-Cas9 with PrimeSTAR (Takara Bio, Shiga, Japan) and purified by phenol/chloroform extraction. The PCR fragments were subjected to *in vitro* mRNA synthesis following same procedure as described above. Sequences of the primers are listed in Supplementary Table S2.

For the syntheses of gRNAs, pDR274-Dma-lig4 vectors were digested by *Dra*I and purified by phenol/chloroform extraction. *Dra*I-digested DNA fragments were used as templates for *in vitro* transcription with the mMessage mMachine T7 kit, followed by column purification with mini Quick Spin RNA columns, phenol/chloroform extraction, ethanol precipitation, and dissolution in DNase/RNase-free water.

#### Donor DNAs

Donor ssODNs were designed to contain the insert comprising 1 nt of cytosine, 60 nt core attP sequences, and 6 nt of the *Bam*HI restriction site, which were located between 40 or 80 nt sequences homologous to the targeted locus. ssODNs were synthesised by Eurofins Genomics (Tokyo, Japan).

To generate the targeting plasmid, 1.5 kb of target-homologous region nearly centred at the TALEN-targeted site was first amplified from genomic DNA by using PrimeSTAR (Takara Bio) and cloned into pCR-BluntII-TOPO vectors (Life Technologies), which resulted in the creation of pCR-BluntII-Dma-ey1.5kb. Second, the 60 bp core attP sequences with *Bam*HI as the insert were generated by PCR from pTA-attP (Addgene plasmid 18939^30^) with KOD plus (TOYOBO, Osaka, Japan). Finally, the attP fragments were cloned into the TALEN-targeted site of the pCR-BluntII-Dma-ey1.5kb by the In-Fusion PCR cloning kit^44^ (Clontech, California, USA). Sequences of the primers are listed in Supplementary Table S2.

### Microinjection

*In vitro* synthesised RNAs and/or donor DNAs were injected into ey^Δ877/Δ1^ mutant eggs according to established procedures^45^. Briefly, eggs were collected from ey^Δ877/Δ1^ daphniids within 2–4 weeks of age just after ovulation and placed in ice-chilled M4 medium containing 80 mM sucrose (M4-sucrose). The synthesised RNAs and/or donor DNAs were injected through a glass needle with N_2_ gas pressure. The injection volume was approximately 0.2 nL. Finally, an injected egg was transferred into each well of a 96-well plate filled with 100 μL of M4-sucrose. Microinjections were carried out within an hour after ovulation. Injections with ey^Δ877/Δ1^ and *Dma-lig4* double mutant eggs were carried out in the same manner as described above.

### Genotyping of G1 offspring

To assess the germ line transmission of site-specific attP integration via HR, we first checked the eye phenotypes of G1 offspring produced by G0 mothers under a stereomicroscope. Then, genome DNAs were extracted from each line of normal eye G1 offspring by previously described procedures with slight modifications^28^. The targeted genomic region was amplified by PCR with TaKaRa Ex Taq HS (Takara Bio, Shiga, Japan). Finally, the PCR products were analysed by agarose gel electrophoresis followed by DNA sequencing.

For *Dma-lig4* mutagenesis, we examined the genotypes of G1 offspring produced by G0 daphniids injected with Cas9 mRNAs and *Dma-lig4* targeting gRNAs. Genome DNAs were extracted from each of 18 randomly selected lines of G1 offspring. The gRNA-targeted genomic region was amplified by PCR TaKaRa Ex Taq HS (Takara Bio, Shiga, Japan). Finally, the PCR products were analysed by polyacrylamide gel electrophoresis followed by DNA sequencing.

To compare the in-del formation rate in the germ line genome between *Dma-lig4* native and mutant strains as described in Fig. 3d,e, genome DNAs were extracted from each of 24 randomly selected lines of G1 offspring from G0 daphniids injected with TALEN mRNAs and donor vector DNA. The targeted genomic region was amplified by PCR with TaKaRa Ex Taq HS (Takara Bio, Shiga, Japan). The PCR products were analysed by polyacrylamide gel electrophoresis. The primers used for PCR and DNA sequencing in this section are listed in Supplementary Table S2.

## Additional Information

**How to cite this article**: Nakanishi, T. *et al.* TALEN-mediated homologous recombination in *Daphnia magna*. *Sci. Rep.* **5**, 18312; doi: 10.1038/srep18312 (2015).

## Supplementary Material

Supplementary Information

## Figures and Tables

**Table 1:** Determining the activity of new TALEN pairs and toxicity of ssODN.

(a) Activity of new TALEN mRNAs						
Injected Construct	Concentration	Embryos	Juveniles	Survival Rate	Deformed eye Juveniles	Phenotypic Changes*
TALEN mRNAs	125 ng/μL	116	86	74%	24/86	28%
	250 ng/μL	95	76	80%	17/76	22%
	500 ng/μL	78	62	79%	26/62	42%

**(b) Toxicity of injecting short ssODN**						
**Injected Construct**	**Concentration**	**Embryos**	**Juveniles**	**Survival Rate**		
Donor	30 ng/μL	42	31	74%		
ssODN	100 ng/μL	44	20	45%		
(107mer)	300 ng/μL	45	0	0%		

*The ratio of phenotypic changes was calculated by the portion of deformed eye juveniles to survived juveniles.

**Table 2:** Efficiencies of attP knock-in with ey^Δ877/Δ1^ strain by TALEN-mediated HR.

Injected constructs	Embryos	Juveniles	Adults		
Donor DNA (homology arm)	Injected	Surviving	Surviving	Revertant lines	Precise attP knock-in lines
vector (1.5 kb)	135	103/135 (76%)	101/135 (75%)	8/101 (7.9%)	2/101 (2.0%)
ssODN (40 nt)	147	113/147 (77%)	88/147 (60%)	7/88 (8.0%)	0/88 (0%)
ssODN (80 nt)	98	64/98 (65%)	52/98 (53%)	8/52 (15%)	1/52 (1.9%)

**Table 3:** Efficiencies of attP knock-in with *Dma-lig4* deficient ey^Δ877/Δ1^ strain by TALEN-mediated HR.

Injected constructs	Embryos	Juveniles	Adults		
Donor DNA (homology arm)	Injected	Surviving	Surviving	Revertant lines	Precise attP knock-in lines
vector (1.5 kb)	61	54/61 (89%)	46/61 (75%)	2/46 (4.3%)	0/46 (0%)
