# Supplementary material for: TALEN-mediated homologous recombination in Daphnia magna
Source: Sci Rep. 2015 Dec 17;5:18312. doi: 10.1038/srep18312 (PMC4682128; doi:10.1038/srep18312)
Supplement: Supplementary Information [file srep18312-s1.pdf]

Supplementary Information

Title

TALEN-mediated homologous recombination in *Daphnia magna*

Authors

Takashi Nakanishi[1], Yasuhiko Kato[1, 2], Tomoaki Matsuura[1], Hajime Watanabe[1, *]

Affiliations

[1]Department of Biotechnology, Graduate School of Engineering, Osaka University, 2-1 Yamadaoka, Suita, Osaka, Japan

[2]Frontier Research Base for Global Young Researchers, Graduate School of Engineering, Osaka University, 2-1 Yamadaoka, Suita, Osaka, Japan

Corresponding author

Hajime Watanabe[*]

E-mail: watanabe@bio.eng.osaka-u.ac.jp

Tel: +81-6-6879-7427, Fax: +81-6-6879-7428

Contents

Supplementary Data S1-4

Supplementary Table S1-2

(a) donor plasmid DNA

5′-CTCTAACGCAGCAGACAACAGCAGCGGAGTAACTTAATGCGGTTATCGGTGTTTCT
GCATGGCTGCACGAAAGGGAAATGCCAAAATGGACTGGCACGACAGCCAATTCCACCCT
CTCTTGCCTCTTTACACGCGCAGCATCTTTTGTGCTGTCACCGGCCAGCGTTGGCCGGG
CAGAGATAAGCTTTTTAAAAGGAAACCCGTTTTTTCAGTATAGCGAGCAAAAGCGTAAC
CCACGCGATGTCGACTCGGTTCGATTTCCCCTCCTTTTTTTTTTCCTTTTTTTCTCTTC
CTTCTTTCATTTTGGAACGACAATAACCAACAGAATATTTTGAAGGGTTTTAGATGATG
ATATACCGAGGCAGTCTATGAAAATGTCCAGATTACGGATAATATATACATGGACGTAT
GTTACATGTGTGTGTGCGTTTTTTTTTTTATATTATAGTCCTTGTGTTTTCGTGTTATT
TTCCTATTACTGCCGCGTTCCTTTGATCTTTGTTGTCGTCTCTCTTAAATGGGTTACTG
TATCTCAGTGATTTATGACGTAGCAACAAATCTCGCTTGGCCAATCAGACGTTATCTTA
AAAACGAAAAAAAAAAAAACATGATTGTTGGTCCTGTCTTTTCAAATTGGCTTTACACG
TATTTATGTATGTTTTTGTTATCTCATTGTAGGCGGTGACGATGTGATGTCGGACGACG
GTGGCGTCAGTTCCGGCGAGAATTCTCGGTCGGCTCCGCCCACGc**cgaccctacgcccc**
caactgagagaactcaaaggttaccccagttggggcactactcccgggatccCGACGAC
AACAACACCTTCGGACGACGATCAGGCTCGACTGCGACTCAAGCGCAAACTCCAGCGCA
ACCGCACTTCCTTCACCAACGAACAGATCGAGAGCCTCGAAAAAGGTGAGCCCCCCAAT
CCGGTGTTTACCCCGTACCGCAACGTCACCATTTGACAATTGGTTTAATTTTTATTTAT
TTATTTTTTAATTTTTTTGGACTATTCTAGAATTTGAACGGACTCATTATCCCGATGTT
TTCGCGCGTGAAAGATTGGCTGCCAAAATTGGACTGCCCGAAGCTCGGATTCAGGTATG
CATTAGTTTCTTCGTAATTCTCTCCTATCCAGGTGGGACAAATCTTTAAAAAAAAAGGC
TAATGATTTTAAACTAATCTTTTACGATGTTATCACAGGTTTGGTTCAGTAACCGACGA
GCAAAATGGCGTCGTGAGGAGAAATTACGGAATCAACGGCGTGGACCAGAACAACAACA
ATCGTCGTCGCAACAGCCGGATCAAGTCAATCCCCAGCAGCAGCAACAACAACAGCAAC
AACAGCAGCTGATCCCTGTGGTTGAACCTGCACCCAGTTCGCCCCGCAGTGTTTCTCAT
TCCGCCAATTATCCCAGTGGCATTTATTCATCTTTGGCCACTACCATGATGACTGATAC
ATACAGGTAATCAATGAATTTAAATTTGACTCTTTATTCCGCGCGGTTTTTAACGGTTT
GCGTTCCCCGTGTTTATCTAAATCCTTTTTTTAGTTCCGCGTTTAATGGAATGAATTCA
ATGAACGGATTTGGTG-3′

(b) short ssODN

5′-CTCGGTCGGCTCCGCCCACGc**cgaccctacgcccccaactgagagaactcaaaggt**
taccccagttggggcactactcccgggatccCGACGACAACAACACCTTCG-3′

(c) long ssODN

5′-CGTCAGTTCCGGCGAGAATTCTCGGTCGGCTCCGCCCACGc**cgaccctacgccccc**
aactgagagaactcaaaggttaccccagttggggcactactcccg<u>ggatcc</u>CGACGACA
ACAACACCTTCGGACGACGATCAGGCTCGACT-3′

(d) partial *Dma-lig4* cDNA sequences

5′-GAAATTGCCTGCATTGGAACAAAAATGGTTAATCAGAATAATCCTAAGAGATGTTA
AACTTACAGGGCTGAGTGACAAGATTGTGTTGTCAGCTTATCATCCAGATGCAAAGGAA
TTATATGATGTCTCAAATGATCTTGAGAAAGTGTGCATAACACTACTTAACCCATTAAG
TCGATCTTCCGAAATAGAAATTTCCCTCTTTAATCCTTTTCGCCCAATGTTAGCAGATA
GACTTGAAATGTCTAAAATTTTCGCGAAAATGGGGAACAAGCCATTCTACGTAGAGACG
AAAATTGACGGAGAAAGAATGCAAATTCATAAAAGCGGAAAGAGGTATGGGTATTATTC
ACGGCGTGGCTTTGACTACACGTCAAACTTCGGAACCAACTCGTCATCGGGAAATCTTA
GCCCTCGTCTAGAGGAATGCTTCCGTGAAACTGTGACCAGTTGCATTATAGATGGTGAA
ATGGTAGCTTGGAACGTCAAAGGTTCCTTTATAGTGTCTAAGGGCGAAAACATAGACGT
CAAGTCCATATCTTCTACTGGCGAACTGATTCCATGTTTCGTCGCTTTTGATATTCTAT
TATTGAATGGCGAAGTCCTATCTAATCTACCATACAAGGAACGACTAAAGCTACTGGAA
TCTGCAGTTAAAATTAAAGAAAGTGTTATTCAGTTTCCGAATCGCAAGCTTGTTACGAC
AAAGATGGAAGTCACTAACTTGCTTAATGACGCAATTGATCAACGCGAAGAAGGAATCG
TCCTGAAAAATCCAGACTCAATCTACAAGCCTAATGAACGCAAAGGAGGATGGTTCAAG
GTCAAACCCGAGTACGTTAACGAAATGATGGATCATTTAGATTTGATCATATTGGGTGG
CTACCATGGACAGGGAAGACGAAGAAATTTGATCGCGATGTTTTTAGTAGGTGTGGCCG
TCCCATCCTTAGAAAAAGAGAAGGAACAACTGGAATTCTATTCTTTCGCTCGTGTTGGC
TC-3′

(e) partial genome sequences on *Dma-lig4* locus from *D. magna* reference genome

5′-ATGGTGTATAATTTTTATGTTTATGCTTTTATGTTTAGGGTACCAAAAACTACACA
AACTCTGGCCAAAGATTTTGCAGACATCTGCTACAACATTTTAAGGACTCGCTGTAGCT
CAGATGTTAGTTCATTGAGTGTTTCTGAAATCAACCAATATCTAGAAAATTTATCAAAA
ACCTACATGTCAAAGAAGTTGGAAAACACAGAAAAGCTTCTGCAGCCTCTCTTGATGAA
ATTGCCTGCATTGGAACAAAAATGGTTAATCAGAATAATCCTAAGAGATGTTAAACTTA
CAGGGCTGAGTGACAAGATTGTGTTGTCAGCTTATCATCCAGATGCAAAGGAATTATAT
GATGTCTCAAATGATCTTGAGAAAGTGTGCATAACACTACTTAACCCATTAAGTCGATC
TTCCGAAATAGAAATTTCCCTCTTTAATCCTTTTCGCCCAATGTTAGCAGATAGACTTG
AAATGTCTAAAATTTTCGCGAAAATGGGGAACAAGCCATTCTACGTAGAGACGAAAATT
GACGGAGAAAGAATGCAAATTCATAAAAGCGGAAAGAGGTTTTAACACCAATGTTCTAA
AAGAAATTTGAGAACTCATATCCACTTCAACAGGTATGGGTATTATTCACGGCGTGGCT

TTGACTACACGTCAAACTTCGGAACCAACTCGTCATCGGGAAATCTTAGCCCTCGTCTA
GAGGAATGCTTCCGTGAAACTGTGACCAGTTGCATTATAGATGGTGAAATGGTAGCTTG
GAACGTCAAAGGTTCCTTTATAGTGTCTAAGGGCGAAAACATAGACGTCAAGTCCATAT
CTTCTACTGGCGAACTGATTCCATGTTTCGTCGCTTTTGATATTCTATTATTGAATGGC
GAAGTCCTATCTAATCTACCATACAAGGTTTTTTTTTCTTCAAAGAACAGTCTTCCGGA
AGAAACTCATTCTATGAATTCTCTTAGGAACGACTAAAGCTACTGGAATCTGCAGTTAA
AATTAAAGAAAGTGTTATTCAGTTTCCGAATCGCAAGCTTGTTACGACAAAGATGGAAG
TCACTAACTTGCTTAATGACGCAATTGATCAACGCGAAGAAGGAATCGTCCTGAAAAAT
CCAGACTCAATCTACAAGCCTAATGAACGCAAAGGAGGATGGTTCAAGGTAAACTGTTG
ATCATTTGAGCTTTCTGTTTAGTGATAAAAATGTATTATTTCTTTAAAGGTCAAACCCG
AGTACGTTAACGAAATGATGGATCATTTAGGTACCGGTCACAAAAAAGAAAGAAATGAA
AAATTAATAGCATTAGTTTCAAATTCAAAAAGTTTTCCAATCTTATGCTACATTAGATT
TGATCATATTGGGTGGCTACCATGGACAGGGAAGACGAAGAAATTTGATCGCGATGTTT
TTAGTAGGTGTGGCCGTCCCATCCTTAGAAAAAGAGAAGGAACAACTGGAATTCTATTC
TTTCGCTCGTGTTGGCTCGGGTTTTTCAGATAAACAACTTCGTAGTTTACTCGAAAAAT
TGAACCCCCATTGGCAAAAGTGGGACAAAAATGCCCCTCCACCAAAAATTCACTGTAGC
AGAGAAAAACCAGATGTTTGGATCAATCCTTCGTCTTCAGCAATATTAGAGGTTAGTTT
GTGTTTATGTTATTAAGAATATGTTTTGTCGAGTTATTCGGGCGCACCATATACGTAGA
TCAAAGCGTCGGAAATTATTTCGAGCAACTCTTACAAAGCCGGATCCACGTTACGATTT
CCCAGAGTGGAGCGCGTACGTGAAGACCGAGCTTGGTATTCCTGTATGACTTTTAGTGA
AATAAACGAAATGAGAAAAATGGCTTCGGGAAAGCTTGTCACGCGCCATTGCAGTTTTC
AAGGTAAAGTTTAGTTTATGCGTTGTTGATGAGTATGGTTGTTTTAAAACTTGTTTTCT
TGTCAATTATTTTGTTTATTGACCCGATTACAGACGATATACCTAGTGGCAAACGCATT
AAAACAGCAAGCGTGATTGCAGGAGTTGCTACTCCCTTCCAAGCAGCAGATATATCTGA
TGTCAAAAGGTCATCAAACTGTTTTTCCGACAAAGAGATTTGCGTTTTAAACGGCTCTA
GTTCCTTTACGAAACAAGAACTAGAACGAAGTGTGGTGTCAGGTGGTGGTGTTGTTGTC
CAGCATCCAAGTGATTTCTTTTTGTACTTTTTTTTTTTGCTGTAGATTTTTTTTTATACC
AAATACTTTTCTTAGCACATTCTACATTTTGCATTATTGCTGCAAAAGACGATATACGA
GTTAAAAGCCTTAAAGCACGACCAAAATGGGATATTGTCTGTCCCGCATGGCTACTGCG
CTGTTTAAACTTGAATCGCCTGATTCCCTTCCGTCCTCAAGATTTACTGGTCGTCACGC
AAGCTACAAAAGAAATACTGGCCCAAAACTTCGATCGATTTGGCAATAGCCTGAGAGAA
GCCACTTCAGTTGAAGATGTTGGTCCGATTCTTCAGCAGGTCAAAGATTTGGTATGGCT
AGTTTTGTATTATTATTTATATATATATACGTATATATATTTTTTTTCGTTCACTTAAG
TAGTTTTTAATTTTTCGTGTGTTAGGAAAACCCGGTGCCTTTAAGTGTATATGAGATAG
CTGATCTTGAATACGAAATATTTGGAAAGTTACACAAGTACGGCATTTTCCGAACCTGC
ATTGTTTATGTCGATGTCTGGGAAACGATATCAGATTTCAATAGCCTAATAATTTCCCG

AGAACTGCGGATCATTTCGCTTGACCTCCGCATCTATGGCGCTCAGGTTTTGAATCACA
TGGACCATTCGGTCACTCATGTTGTATGTGATCCAGTCGCTTACCCTCATCGAGCCTCT
ATGTGGAAATCGATAAATCGTCAGCAAGAAGTCAAGTTTAAATTAGTTCGACCAGAGTG
GATTGCACATTCCATAACCCAAGGGAATTTGCTTGATGAAATAATCTATTGTCCCTAG-3′

Supplementary Data S1. Sequences of donor DNAs and *Dma-lig4* gene

Sequences of donor DNAs and *Dma-lig4* gene. (**a-c**) DNA sequences of donor DNAs are shown. For donor plasmid DNA, only sequences of homology arms and 67 bp insert DNA containing attP are described. Uppercase indicates the homology arms, whereas lowercase indicates the exogenous insert DNA sequences in which the core 60 bases of attP are highlighted in bold and 6 bases of the *Bam*HI site are underlined. (**d**, **e**) DNA sequences of *Dma-lig4* cDNA (**d**) and reference genome (**e**) are shown.

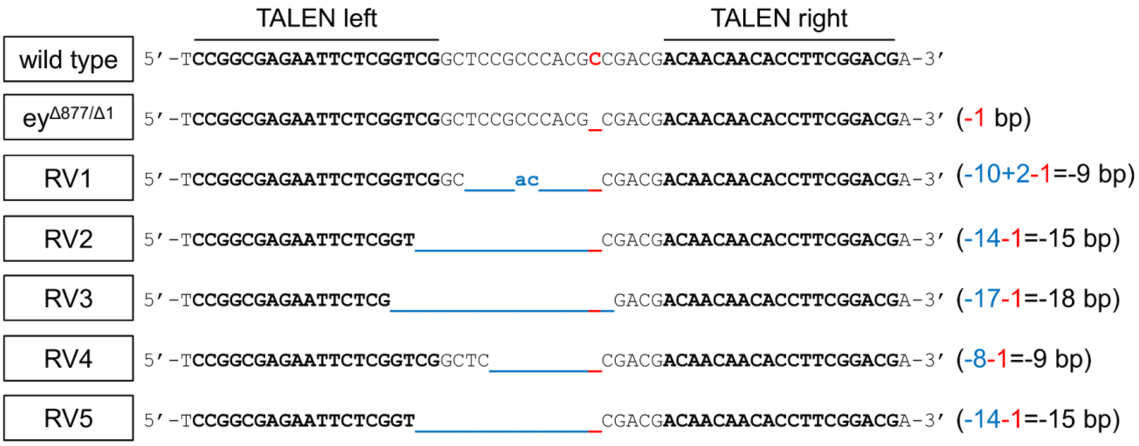

Supplementary Data S2. Genotypes of G1 revertant offspring generated by additional in-dels

Genome sequences around the TALEN targeted sites on the *Dma-ey* locus among revertant (RV) offspring generated by additional in-dels. TALEN-binding DNA sequences are highlighted in bold. The deleted base ‘C’ on the ey$^{\Delta877/\Delta1}$ genome is highlighted in red. Additional in-dels induced by TALENs are highlighted in blue. The total number of in-dels is calculated on the right side. Note that all RVs had multiple 3 in-dels in total, which led to the recovery of the *Dma-ey* reading frame. RV1 is derived from co-injection of TALEN mRNAs and short ssODN, RV2 and 3 are from co-injection of TALEN mRNAs and long ssODN, and RV4 and 5 are from co-injection of TALEN mRNAs and donor plasmid DNA.

```
Dmagna_LIG4    1 ------------------------------------------------------------
human__LIG4    1 MAASQTSQTVASHVPFADLCSTLERIQKSKGRAEKIRHFREFLDSWRKFHDALHKNH---
Dmelan_LIG4    1 -----MSVDIASTIKFRDICSLFEKIKATQKVANKEEVLKSYYESFCRHRESFRRQTGLN
yeast__DNL4    1 --MISALDSIPEPQNFAPSPDFKWLCEELFVKIHEVQINGTAGTGKSRSFKYYEIISNFV

Dmagna_LIG4    1 ----------------------------------MVYNFYVYAF-------------MFR
human__LIG4   58 ----KDVTDSFYPAMRLILPQLERERMAYGIKETMLAKLYIELLNLPRDGKDALKLLNYR
Dmelan_LIG4   56 NDQPEDGASSFYSVLRLLLPGADTGRDTYGLQITALGRLYIRVLQLPTDSSD-----AIR
yeast__DNL4   59 EMWRKTVGNNIYPALVLALP--YRDRRIYNIKDYVLIRTICSYLKLPKNSATEQRLKDWK

                           <----------------DNA_ligase N terminus domain-----
Dmagna_LIG4   14 VPKTTQTLAKDFADICYNILRTRCSSDVSSLSVSEINQYLENLSKTYMSKKLENT----E
human__LIG4  114 TPTGTHGDAGDFAMIAYFVLKPRCLQKGS-LTIQQVNDLLDSIASNNSAKRKDLI----K
Dmelan_LIG4  111 LQHRNGNMYRDYGDVVYSVLKPRCFNPPSNLRLKEIHQMLDTIAN----EDTEVK----Q
yeast__DNL4  117 QRVGKGGNLS--SLLVEEIAKRRAEPSSKAITIDNVNHYLDSLSGDRFASGRGFKSLVKS

                 -----------------------------------------&gt;
Dmagna_LIG4   70 KLLQPLLMKLPALEQKWLIRIILRDVKLTGLSDKIVLSAYHPDAKELYDVSNDLEKVCIT
human__LIG4  169 KSLLQLITQSSALEQKWLIRMIIKDLKLG-VSQQTIFSVFHNDAAELHNVTTDLEKVCRQ
Dmelan_LIG4  163 QQLIRFTEQASPEEQKWLIRLLLKSLGLG-IGEQKIFGVLHPKAQDIYQRCSDLGHVCNL
yeast__DNL4  175 KPFLHCVENMSFVELKYFFDIVLKNRVIG-GQEHKLLNCWHPDAQDYLSVISDLKVVTSK

                          &lt;--------------------------------------------------
Dmagna_LIG4  130 LLNPLSRSSE----------------IEISLFNPFRPMLADRLEMS-KIFAKMGNKPFYV
human__LIG4  228 LHDPSVGLSD----------------ISITLFSAFKPMLAAIADIE-HIEKDMKHQSFYI
Dmelan_LIG4  222 LADRTTDLDASSSKDSKAAVKFVNLNSVIRPFHQIRPMLCERFPG--DIQELMQSDVLYL
yeast__DNL4  234 LYDPKVRLKDD--------------DLSIKVGFAFAPQLAKKVNLSYEKICRTLHDDFLV

                 ---------------------------Adenylation domain---------------
Dmagna_LIG4  173 ETKIDGERMQIHKS--GKRYGYYSRRGFDYTSNFGTNSSSGNLSPRLEECFRETVTSCII
human__LIG4  271 ETKLDGERMQMHKD--GDVYKYFSRNGYNYTDQFGASPTEGSLTPFIHNAFKADIQICIL
Dmelan_LIG4  280 ETKMDGERFQLHID--RGRFMYISRNGVDYTRNFGHSYDHGTLTPKLRGLLPLGLESIIL
yeast__DNL4  280 EEKMDGERIQVHYMNYGESIKFFSRRGIDYTYLYGASLSSGTISQHLR--FTDSVKECVL

                 ------------------------------------------------------------
```

```
Dmagna_LIG4  231 DGEM--------------------------------------------------------
human__LIG4  329 DGEMMAYNPNTQTFMQK------GTKFDIKRMVEDSDLQTCYCVFDVLMVNNKKLGHETL
Dmelan_LIG4  338 DGEMMVWDTNKLRFREK------GENTDVKSLKPEGSWQPCFVVYDLLYFNGQSLLDHTY
yeast__DNL4  338 DGEMVTFDAKRRVILPFGLVKGSAKEALSFNSINNVDFHPLYMVFDLLYLNGTSLTPLPL

                 ------------------------------------------------------------
Dmagna_LIG4  235 -ERLKLLESAVKIKESVIQFPNRKLVTTKMEVTNLLNDAIDQREEGIVLKNPDSIYKPNE
human__LIG4  383 RKRYEILSSIFTPIPGRIEIVQKTQAHTKNEVIDALNEAIDKREEGIMVKQPLSIYKPDK
Dmelan_LIG4  392 IQRAYKLQKLIVEQSGVLQLMRARKIGSVQEFNELFQQALDSHAEGIVLKKQGSRYQPGV
yeast__DNL4  398 HQRKQYLNSILSPLKNIVEIVRSSRCYGVESIKKSLEVAISLGSEGVVLKYYNSSYNVAS

                 ------->                        <---------------------------
Dmagna_LIG4  294 RKG-GWFK------------------------GRRRNLIAMFLVGVAV------------
human__LIG4  443 RGE-GWLKIKPEYVSGLMDELDILIVGGYWGKGSRGGMMSHFLCAVAE------------
Dmelan_LIG4  452 RLGGGWYKDKADYIKGLITEFDVLIIG--AFYNRKRTFVDSFLLGVLQ------------
yeast__DNL4  458 RNN-NWIKVKPEYLEEFGENLDLIVIG--RDSGKKDSFMLGLLVLDEEEYKKHQGDSSEI

                 -----------Oligonucleotide binding fold domain--------------
Dmagna_LIG4  317 --------PSLEKEKEQLEFYSFARVGSGFSDKQLRSLLEKLNPHWQKWDKNAPPPKIHC
human__LIG4  490 --------KPPPGEK-PSVFHTLSRVGSGCTMKELYDLGLKLAKYWKPFHRKAPPSSILC
Dmelan_LIG4  498 --------PAPPGSSNRPEVFSIGVVAN--NTKQRGVLNHTLKPHWHDVVNEPPPLWFHY
yeast__DNL4  515 VDHSSQEKHIQNSRRRVKKILSFCSIANGISQEEFKEIDRKTRGHWKRTSEVAPPASILE

                 ------------------------------------------------------------
Dmagna_LIG4  369 SREK----PDVWINPSSSAILEIKASEIISSNS----YKAGSTLRFPRVERVREDRAWYS
human__LIG4  541 GTEK----PEVYIEPCNSVIVQIKAAEIVPSDM----YKTGCTLRFPRIEKIRDDKEWHE
Dmelan_LIG4  548 KPKERSGCPDLWIEPQNSVILQVKAADIAPNGA----FFTRKSLHFPRTEMKRDDKTWSE
yeast__DNL4  575 FGSK---IPAEWIDPSESIVLEIKSRSLDNTETNMQKYATNCTLYGGYCKRIRYDKEWTD

                 --->
Dmagna_LIG4  421 CMTFSEINEMRKMASGKLVTRHCSFQ-DDIPSGKR-IKTAS---VIAGVATPFQAADISD
human__LIG4  593 CMTLDDLEQLRGKASGKLASKHLYIGGDDEPQEKK-RKAAPKMKKVIGIIEHLKAPNLTN
Dmelan_LIG4  604 CMTLKEFNDLCGGPLAIKKLNKRQLRLEDVTTKRKQMRMTPSERSRLGLAVYEKRYDAST
yeast__DNL4  632 CYTLNDLYESRTVKSNPSYQAERSQLGLIRKKRKR---------VLISDSFHQNRKQLPI
```

```
                     <-----------------BRCT domain---------------------------
Dmagna_LIG4  476 VKRSS-NCFSDKEICVLNGSS-SFTKQELERSVVSAH--------------STFCIIAAK
human__LIG4  652 VNKIS-NIFEDVEFCVMSGTD-SQPKPDLENRIAEFGGYIVQNPGP-----DTYCVIAGS
Dmelan_LIG4  664 SASTS-KLFDGLSFCILSGSAGRHSKHQLQELAVKNGGCIVENPLPN---DPKCFCIAGD
yeast__DNL4  683 SNIFAGLLFYVLSDYVTEDTGIRITRAELEKTIVEHGGKLIYNVILKRHSIGDVRLISCK

                 ----------------------->
Dmagna_LIG4  520 DDIRVKSLKARP--KWDIVCPAWLLRCLNLNRLIPFRPQDLLVVTQATKEILAQNFDRFG
human__LIG4  705 ENIRVKNIILSN--KHDVVKPAWLLECFKTKSFVPWQPRFMIHMCPSTKEHFAREYDCYG
Dmelan_LIG4  720 ETFLVKRLILQQPRTCDIVRMEWLLRVCQKQ-ELELKPRDLIAATEPLQQDLAECFDRHG
yeast__DNL4  743 TTTECKALIDRG---YDILHPNWVLDCIAYKRLILIEPNYCFNVSQKMRAVAEKRVDCLG

Dmagna_LIG4  578 NSLREATS-VEDVGPILQQVKDLENPVPLSVY-EIADLEYEIFGKLHKYGIFRTCIVYVD
human__LIG4  763 DSYFIDTD-LNQLKEVFSGIKNSNEQTPEEMASLIADLEYRYSWDCSPLSMFRRHTVYLD
Dmelan_LIG4  779 DSYTKDIANVEELQDLLQGIELTADNVAGITASNLNALEDQLLDGKKNLNMFRNLNAFFY
yeast__DNL4  800 DSFENDIS----------ETKLSSLYKSQLSLPPMGELEIDSEVRRFPLFLFSNRIAYVP

                                       <------------BRCT domain-------------
Dmagna_LIG4  636 VWETISDFNSLIISRELRIISLDLRIYGAQVLNHMDHSVTHVVCDPV---------AYPH
human__LIG4  822 SYAVINDLSTKNEGTRLAIKALELRFHGAKVVSCLAEGVSHVIIG-----------EDHS
Dmelan_LIG4  839 S-----------PHGDEVAKLLFLQNGGRIVDDSDPQLNLGFICMSS---------DIDN
yeast__DNL4  850 R---------RKISTEDDIIEMKIKLFGGKITDQQSLCNLIIIPYTDPILRKDCMNEVHE

                 ---------------------------->
Dmagna_LIG4  687 RASMWKSINRQQEVKFKLVRPEWIAHSITQGNLLDEIIYCP---
human__LIG4  871 RVADFKAFRRTFKRKFKILKESWVTDSIDKCELQEENQYLI---
Dmelan_LIG4  879 DHFEHWLHNHSKLTTDKVLNSAWIHQCHREGILLPMHSFV----
yeast__DNL4  901 KIKEQIKASDTIPKIARVVAPEWVDHSINENCQVPEEDFPVVNY
```

Supplementary Data S3. Alignment of deduced Dma-LIG4 amino acid sequences with human, fly and yeast LIG4 proteins

Sequence alignment of *Daphnia magna* LIG4, human LIG4 (Accession: NP002303), *Drosophila melanogaster* LIG4 (NP572907), and yeast (*Saccharomyces cerevisiae*) DNL4 (NP014647). Protein sequences were aligned by the ClustalW algorithm (http://clustalw.ddbj.nig.ac.jp/). Identical and similar residues are shown by black and grey backgrounds, respectively, using Boxshade program. The Dma-LIG4 protein

shows high sequence homology with human, *Drosophila*, and yeast DNL4 proteins (30, 27, and 22% identity, respectively). The active residue lysine at position 175 is highlighted by a red background. LIG4 functional domains, such as DNA ligase N terminus domain (Accession: pfam04675), adenylation domain (cd07903), oligonucleotide binding fold domain (cd07968), and BRCT domain (cd00027), are all conserved in Dma-LIG4, and are indicated on the top line of alignment.

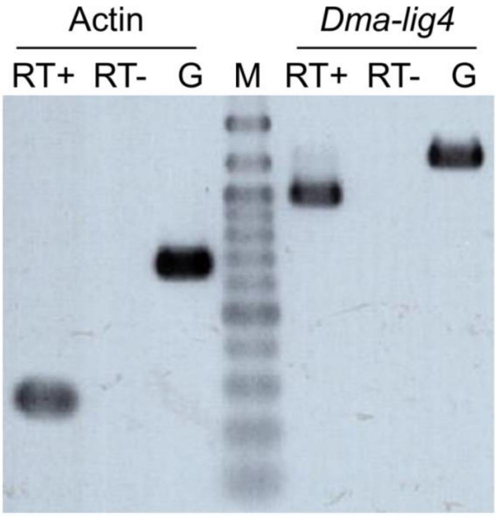

Supplementary Data S4. RT-PCR of the *Dma-lig4* gene

Agarose gel electrophoresis of RT-PCR products amplified from *Actin* and *Dma-lig4* genes. M: marker DNA, G: genomic PCR product as a positive control of PCR, RT+: RT-PCR product amplified from reverse-transcribed cDNAs, RT-: RT-PCR product amplified from total RNAs without reverse transcription.

Supplementary Table S1. Details of microinjection experiments

Injected constructs	Replicate	Embryos	Juveniles	Adults	Revertant	HR knock-in
TALEN mRNAs, targeting plasmid	Exp. 1	47	33	33	4	1
	Exp. 2	36	25	24	1	0
	Exp.3	52	45	44	3	1
	total	135	103	101	8	2
TALEN mRNAs, short ssODN	Exp. 1	52	41	26	3	0
	Exp. 2	14	9	8	0	0
	Exp. 3	31	27	19	1	0
	Exp. 4	50	36	35	3	0
	total	147	113	88	7	0
TALEN mRNAs, long ssODN	Exp. 1 (=total)	98	64	52	8	1

Supplementary Table S2. List of oligonucleotides used in this study

A. *Dma-lig4* targeting constructs

Name	Target site (5′–3′, PAM shown by lowercase)	Sense oligonucleotide (5′–3′)	Antisense oligonucleotide (5′–3′)
gRNA_Dma-lig4_1	AGCTTATCATCCAG ATGCAAagg	TAGGCTTATCATCC AGATGCAA	AAACTTGCATCTGG ATGATAAG
gRNA_Dma-lig4_2	GGAAGCATTCCTCT AGACGAggg	TAGGAAGCATTCCT CTAGACGA	AAACTCGTCTAGAG GAATGCTT

B. Primers

Name	Sequence (5′–3′)	Purpose
Dma-ey_homeo_fwd3	CTCTAACGCAGCAGACAACAG	Construction of donor plasmid
Dma-ey_homeo_rev	CACCAAATCCGTTCATTGA	
IF-ey1.5k-fwd	CGACGACAACAACACCTTCGGACG	
IF-ey1.5k-rev	GCGTGGGCGGAGCCGACC	
IF-eyattP-fwd	CGGCTCCGCCCACGCCGACCCTACG CCCCCAAC	
IF-eyBamHIattP-rev	GTGTTGTTGTCGTCGGGATCCCGGG AGTAGTGCCCCAAC	
Dma-ey_homeo_fwd	GTGACGATGTGATGTCGGA	Genotyping and Sequencing on Dma-ey locus
Dma-ey_homeo_rev4	GAGGCTCTCGATCTGTTCG	
Dma-lig4-RT-fwd	GAAATTGCCTGCATTGGAAC	Genotyping and Sequencing on Dma-lig4 locus
Dma-lig5-RT-rev	GAGCCAACACGAGCGAAAG	
Dma-lig4-genome-fwd1	TATGGGTATTATTCACGGCG	
Dma-lig4-genome-rev2	TCCAAGCTACCATTTCACCA	
T7-vasCas9-IVT-fwd	TAATACGACTCACTATAGGGAGGAG ACAAAAC	Template synthesis for Cas9 mRNA transcription
Dmavas-3UTR-rev	AACAAAATGAATTCGTTCTGTATTC	
